# Fecal Microbial Signatures Are Associated With Engraftment Failure Following Umbilical Cord Blood Transplantation in Pediatric Crohn’s Disease Patients With *IL10RA* Deficiency

**DOI:** 10.3389/fphar.2020.580817

**Published:** 2020-10-08

**Authors:** Aijuan Xue, Xiaowen Qian, Xuefeng Gao, Ping Wang, Lin Wang, Cuifang Zheng, Zhiheng Huang, Wenhui Hu, Jieru Shi, Ying Huang

**Affiliations:** ^1^Department of Gastroenterology, Children’s Hospital of Fudan University, Shanghai, China; ^2^Department of Hematology, Children’s Hospital of Fudan University, Shanghai, China; ^3^Department of Hematology-Oncology, International Cancer Center, Shenzhen University General Hospital, Shenzhen University Health Science Center, Shenzhen, China

**Keywords:** Crohn’s disease, *IL10RA*, pediatric, microbiome, umbilical cord blood transplantation

## Abstract

**Objectives:**

Umbilical cord blood transplantation (UCBT) is associated with a relatively high rate of engraftment failure. This study aimed at exploring whether any fecal microbiota could be associated with engraftment failure following UCBT in Crohn’s disease patients with *IL10RA* deficiency.

**Methods:**

Thirteen patients were recruited and their 230 fecal samples were collected longitudinally from immediately before conditioning chemotherapy to 8 weeks post the UCBT. The V3-V4 regions of the bacterial 16S rRNA gene were amplified by PCR and sequenced, followed by bioinformatics analyses.

**Results:**

Following the UCBT, 7 out of 13 patients achieved neutrophil and platelet engraftment with a median of 21 and 28 days, respectively (S group), while 6 patients failed to achieve engraftment (F group). In comparison with that in the S group, significantly lower Shannon diversity values on the UCBT day (*P* = 0.0176) and less abundance of *Bifidobacterium longum*, *Bifidobacterium pseudolongum*, *Enterobacteriaceae_538000*, and one taxon of *Lachnospiraceae* family was detected in the F group, accompanied by significantly higher abundances of four taxa including *Lautropia*, *Pseudomonas*, and species *Microvirgula aerodenitrificans* during the chemotherapy period as well as UCBT. The abundances of thirty OTUs were correlated significantly with clinical indices.

**Conclusions:**

Microbial indicators of reduced diversity of microbiota and signatures of specific bacterial abundances, such as a lower abundance of *Bifidobacterium longum*, for engraftment failure would require validation. These indicators may help for the risk stratification in patients with *IL10RA* deficiency undergoing UCBT.

## Introduction

Inflammatory bowel diseases (IBD) are chronic relapsing inflammatory disorders in the gastrointestinal tract, and classified into Crohn’s disease (CD), ulcerative colitis (UC), and IBD-undefined. IBD can affect different ages of subjects, including infants, who usually have genetic mutations, such as the *IL10RA* gene, the most common genetic factor of IBD in China (Huang et al., 2017; Ye et al., 2017). Pediatric IBD patients, particularly for CD patients, usually have severe symptoms (Ye et al., 2017). Pathologically, their colonic tissues are affected, accompanied by multiple perianal diseases. These, together with resistant to standard medical therapies, lead to a high mortality in China (Huang et al., 2017; Zheng et al., 2019).

Hematopoietic stem cell (HSC) transplantation is emerging as an alternative therapeutic approach for the treatment of IBD (Khalil et al., 2007). Currently, several clinical trials of HSC treatment for CD have been reported (Hawkey, 2012). Umbilical cord blood is a rich source of hematopoietic stem cells, and umbilical cord blood transplantation (UCBT) has been used for treatment of malignant and nonmalignant diseases to re-establishment of the hematopoietic system and correct immunodeficiency (Frassoni et al., 2003; Munoz et al., 2014)._ENREF_5 Previous studies have shown that UCBT with reduced-intensity conditioning has represented a curable treatment for patients with *IL10RA*-associated immune deficiency, making it a potential treatment for IBD (Uhlig and Muise, 2017; Ye et al., 2017; Peng et al., 2018; Zheng et al., 2019). Unfortunately, some IBD patients with *IL10RA* deficiency fail to achieve successful engraftment after the UCBT,_ENREF_3 limiting the application of UCBT in IBD patients (Zheng et al., 2019). However, the impact of defective IL10 signaling on human intestinal inflammation has not been clarified (Glocker et al., 2009; Zheng et al., 2019) and little is known on risk factors associated with engraftment failure after the UCBT in IBD patients with *IL10RA* deficiency.

It is well known that many factors, such as high number of HLA mismatches and low doses of cord blood cells, are associated with inferior transplant outcomes (Rocha, 2016; Kiernan et al., 2017). However, little is known about the potential role of gut microbiota dysbiosis in the UCBT outcomes. Recent studies have shown that allo-hematopoietic stem cell transplantation (allo-HSCT) can alter the components and diversity of gut microbiota in rodents and humans (Wang et al., 2015; Peled et al., 2017; Shallis et al., 2018; Shono and van den Brink, 2018; Ingham et al., 2019). Reciprocally, the intestinal microbiota can regulate hematopoiesis and promote myeloid differentiation (Shono and van den Brink, 2018). Intestinal bacteria are potent modulators of systemic immune responses (Zama et al., 2020). Alteration in the intestinal microbiota is associated with graft-versus-host disease (GVHD), bacteremia, disease relapse, and mortality after allo-HSCT. Furthermore, distinct gut microbial profiles were speculated to be biomarkers for diagnosis of allo-HSCT-related complications (Shallis et al., 2018). However, there is no information on whether a unique gut microbial profile can predict the engraftment failure of the UCBT in IBD patients with *IL10RA* deficiency.

Given that altered microbiota affect hematopoiesis and immune reconstitution in allo-HSCT recipients, modulation of intestinal microbiota may promote healthy post-transplant immunity. Accordingly, it is critical to define the unique intestinal bacterial profiles associated with engraftment failure to predict and prevent engraftment failure in IBD patients with *IL10RA* deficiency after the UCBT. In this study, we monitored the dynamic changes in engraftment and the intestinal microbiome longitudinally in 13 CD patients with *IL10RA* deficiency throughout the UCBT course to establish the preliminary association between intestinal microbial profiles and engraftment failure in these patients.

## Materials and Methods

### Ethical Consideration

This study was approved by the Ethical Committee of Children’s Hospital, Fudan University (Shanghai, China, ethics no. [2017]-230). Oral or written informed consent was obtained from individual parents or their guardians prior to sample collection.

### Subjects and Data Collection

A total of 17 *IL10RA* deficient patients (age: 0–18 years) with CD and receiving UCBT were enrolled at the Children’s Hospital of Fudan University (China) between October 1, 2017 and December 31, 2018. Individual patients with CD were diagnosed, according to the revised Porto criteria for the diagnosis of PIBD (Levine et al., 2014). The inclusion criteria included (i) receiving UCBT from a regularly delivered newborn between October 2017 and December 2018; (ii) without experience of ostomy and extensive bowel resection; (iii) formed or semi-formed stool for sampling. Their demographic and clinical data were obtained from medical records, including age, gender, bodyweight, age at onset, medical history, disease activity, and laboratory results. The laboratory tests included serum C-reactive protein (CRP), blood hemoglobin levels, platelet, and neutrophil counts.

### RIC Chemotherapy and UCBT Procedure

All eligible patients undergoing UCBT received the same reduced-intensity conditioning (RIC) regimens, including fludarabine, busulfan and cyclophosphamide beginning on day -9. They received pre-medications and hydration prior to UCB infusion (day 0), as described previously (Peng et al., 2018). Those patients were provided with prophylactic and therapeutic antibiotics, as a routine clinical practice.

### Engraftment

Engraftment was defined as the first 3 consecutive days with an absolute neutrophil count of ≥0.5 × 10^9^/L, and the first 7 consecutive days with a platelet count of ≥20 × 10^9^/L without platelet transfusion (Popat et al., 2015).

### Fecal Sample Collection and DNA Extraction

Fecal samples were collected daily beginning on day -10 for 17 consecutive days (from one day before the RIC chemotherapy to day 7 post the UCBT), and then weekly for three times and biweekly for two times post the UCBT. All fecal samples were stored at -80°C immediately after collection.

Total DNA of individual fecal samples was extracted using the standardized IHMS protocol Q recommended by the International Human Microbiome Consortium (IHMS_SOP_06_V1). Briefly, total DNA in individual fecal samples was extracted using the QIAamp DNA Stool Mini Kit (Qiagen, Germany) and bead-beating with 0.5 g of autoclaved zirconium beads (0.1 mm, BioSpec Products, Bartlesville, OK) in a Scientz‐48 High-throughput tissue grinder (Scientz, China). The extracted DNA was used as the templates for in triplicate amplification of the V3-V4 variable regions of the bacterial 16S rRNA gene using barcoded primers by PCR. The PCR products were purified using AMPure XP beads (Beckman Coulter, Brea, California), and equal amount of products from each sample was pooled to form a library. After quantification and qualification using Agilent Bioanalyzer 2100 (Agilent Technologies, USA), the DNA samples were sequenced on a Novaseq 6000 platform (Illumina, San Diego, CA, USA) using the 2 × 250 bp paired end protocol.

### 16 rRNA Gene Analysis

The raw sequencing data were processed using QIIME1 (version 1.9) (Caporaso et al., 2010). The paired reads were merged using *join_paired_ends.py* command. The merged sequences were demultiplexed using *split_libraries_fastq.py* command at Phred ≥ Q20. The unique sequences were clustered into operational taxonomic units (OTUs) using *pick_closed_reference_otus.p*y against the Greengenes database (version 13.8) at 97% identity (DeSantis et al., 2006). The OTU abundances were normalized by total-sum, cumulative-sum, and log 2 transformation using Calypso version 8.84 (Zakrzewski et al., 2017). Alpha diversity at the OTU level of each group was analyzed using Shannon diversity index [1-E(H)]. Given a vector of OTU counts, the Shannon diversity index is computed as $H=\frac{nlog\left(n\right)-\sum_{i-1}^{k}f_{i}\text{log}\left(f_{i}\right)}{n}$ with *k* and *n* denoting the number of groups and the total counts, respectively. The Shannon equitability index *E(H)* was the Shannon diversity index divided by the maximum diversity $E\left(H\right)=\frac{H}{\text{log}\left(k\right)}$. The Beta-diversity in bacterial communities was estimated by principal coordinate analysis (PCoA), based on Bray-Curtis distance. The difference between the relative abundances of alpha diversity or taxa between two groups was determined by the Wilcoxon rank-sum test with the Benjamini-Hochberg procedure. The associations between the relative abundances of gut bacteria and serum CRP, blood hemoglobin levels, platelet and neutrophil counts were analyzed by Spearman rank correlations. The microbial phenotypes at an organism level were predicted using BugBase (Ward et al., 2017).

## Results

### Demographic and Clinical Characteristics of Patients and Their Fecal Sample Collection

To determine the potential association of gut microbiota with the outcomes of UCBT in CD, 17 *IL10RA*-deficient CD patients undergoing UCBT were assessed, 13 patients were included and their 230 stool samples were collected (13–22 samples per patient). Four patients were excluded due to ostomy (N = 2) or inadequate stool samples (N = 2). Following the UCBT, 7 out of 13 patients achieved neutrophil and platelet engraftment with a median of 21 and 28 days, respectively (S group), while the remaining 6 patients failed to achieve engraftment (F group). There was no significant difference in age, gender, body weights, BMI, disease duration, disease severity, laboratory results and medications at their admission for the UCBT (**Table 1**). In comparison with that in the F group, the median neutrophil counts (*P* = 0.0003) and blood hemoglobin levels (*P* = 0.0004) were significantly higher in the S group during the RIC chemotherapy and after the transfusion, respectively. In contrast, there was no significant difference in the median platelet counts and GVHD rates between these two groups during the observation period. While there was no patient death in the S group, five out of six patients in the F group died of the UCBT-related severe complications, such as respiratory failure (N = 2) and sepsis (N = 3). The extensive details of individual patients are shown in **Table S1**.

**Table 1:** Characteristics of *IL10RA*-deficiency patients receiving umbilical cord blood transplantation.

Variable	S group (n = 7)	F group (n = 6)	P value
**Pre-transplant characteristic**			
Median age, month	13(5–26)	11.5(7–28)	0.67
Female (%)	4(57)	2(33)	0.59
Median weight, kg	7.5(3.7–12)	6.85(5–11)	0.89
Median BMI, kg/m2	15.22(11.80–18.77)	16.16(13.81–19.84)	0.32
Disease duration, month	12(4–17)	11.5(6–28)	0.57
CRP<8 mg/L (%)	4(57)	3(50)	1.00
Median hemoglobin, g/L	93(75–104)	96(86–133)	0.47
Median PLT	461(337–771)	438(381–514)	0.88
Median neutrophil count, 10^9^	3.3(1.82–13.08)	1.88(1.82–3.01)	0.23
wPCDAI	45(12.5–65)	37.5(5–55)	0.57
Medications			
Mesalazine (%)	6(86)	6(100)	1.00
Thalidomide (%)	7(100)	6(100)	1.00
Steroids (%)	3(43)	1(17)	0.5
Proton pump inhibitors (%)	2(29)	0(0)	0.46
**During chemotherapy**			
Median hemoglobin, g/L	91(75–116)	97(75–121)	0.06
Median PLT	444(272–898)	402(207–871)	0.07
Median neutrophil count, 10^9^	3.39(0.52–11.33)	1.75(0.34–3.21)	0.0003
**After the UCBT**			
Median hemoglobin, g/L	93(69–119)	83.5(13–123)	0.0004
Median PLT	134(2–564)	104(2–419)	0.31
Median neutrophil count, 10^9^	0.65(0.01–7.52)	0.42(0.06–8.49)	0.76
PLT engraftment, day	28(17–35)	FE	NA
Neutrophil engraftment, day	21(13–31)	FE	NA
GVHD	4 (57)	2 (33)	0.59
Alive (%)	7(100)	1(17)	0.005

Variables are presented as median (range) unless otherwise indicated. Plt, platelets count; FE, failure to engraft; NA, not applicable.

### The Dynamic Changes in Microbial Diversity

Analysis of the gut microbiota profiles with 16S rRNA gene sequencing revealed considerable variations of the gut microbiota alpha diversity (a wide interquartile range) in the F group during the chemotherapy period (**Figure S1**). At the baseline, there was a slightly higher enrichment in the gut microbiota (measured by Chao1 index) of F group prior to chemotherapy (**Figure S2**). During the chemotherapy and post-transplant periods, the overall alpha diversity in both groups fluctuated without a clear trend (*P* > 0.05). Although the average Shannon diversity values between these two groups were similar, a hallmark of relatively stable during the chemotherapy and UCBT, the average alpha diversity on the transplant day (T0d) in the F group was significantly lower than in the S group (*P* = 0.0176).

**Figure 1:**
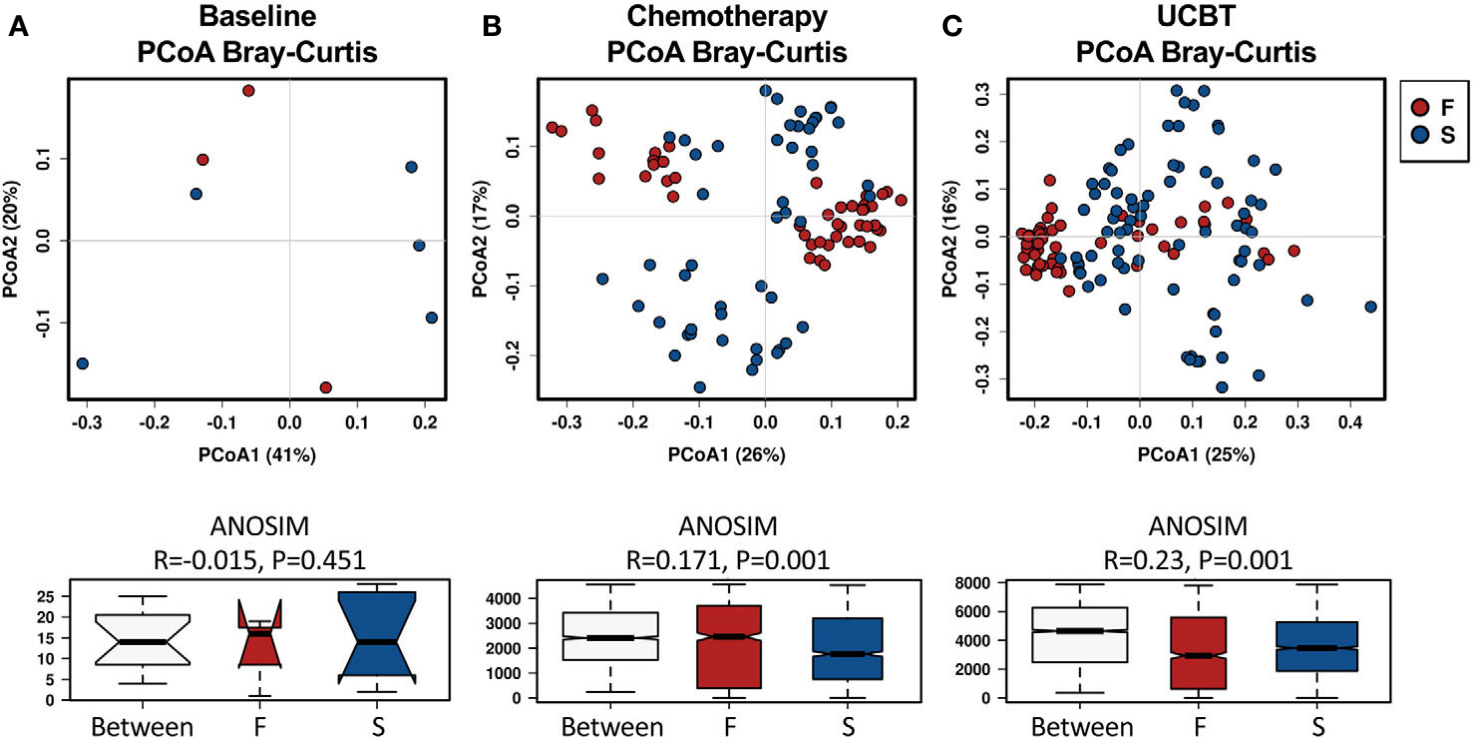
Principal coordinate analysis (PCoA) of the beta-diversity differences based on Bray-Curtis distances between the F and S groups during the treatment course. Groups were indicated in different colors. **(A)** Baseline. **(B)** Chemotherapy. **(C)** During UCBT.

Analysis of the gut microbiota beta diversity with PCoA revealed a significant difference in the Bray-Curtis distances between the F and S group. Interestingly, fecal microbiome in the F group clustered closer than in the S group, indicating that fecal microbiota from different individuals in the F group were similar during the chemotherapy and after the UCBT (**Figure 1**). Such results were also reproduced by PCoA with Unweighted Unifrac or Weighted Unifrac distance (**Figure S3**).

### Longitudinal Characteristics of Gut Microbiome in UCBT-Treated CD

To evaluate alterations in the gut microbiome throughout the UCBT, we analyzed the longitudinal changes in the relative abundance of fecal bacteria. Compositionally, *Proteobacteria* and *Firmicutes* were the dominant phyla in both groups (**Figures S4A, B**). Among the top 12 most prevalent genera in each group, *Clostridium*, *Enterococcus*, *unclassified. Enterococcaceae*, *Lactococcus*, *Staphylococcus*, *Streptococcus*, and *Vagococcus* were shared by both groups (**Figures S4C, D**).

**Figure 2:**
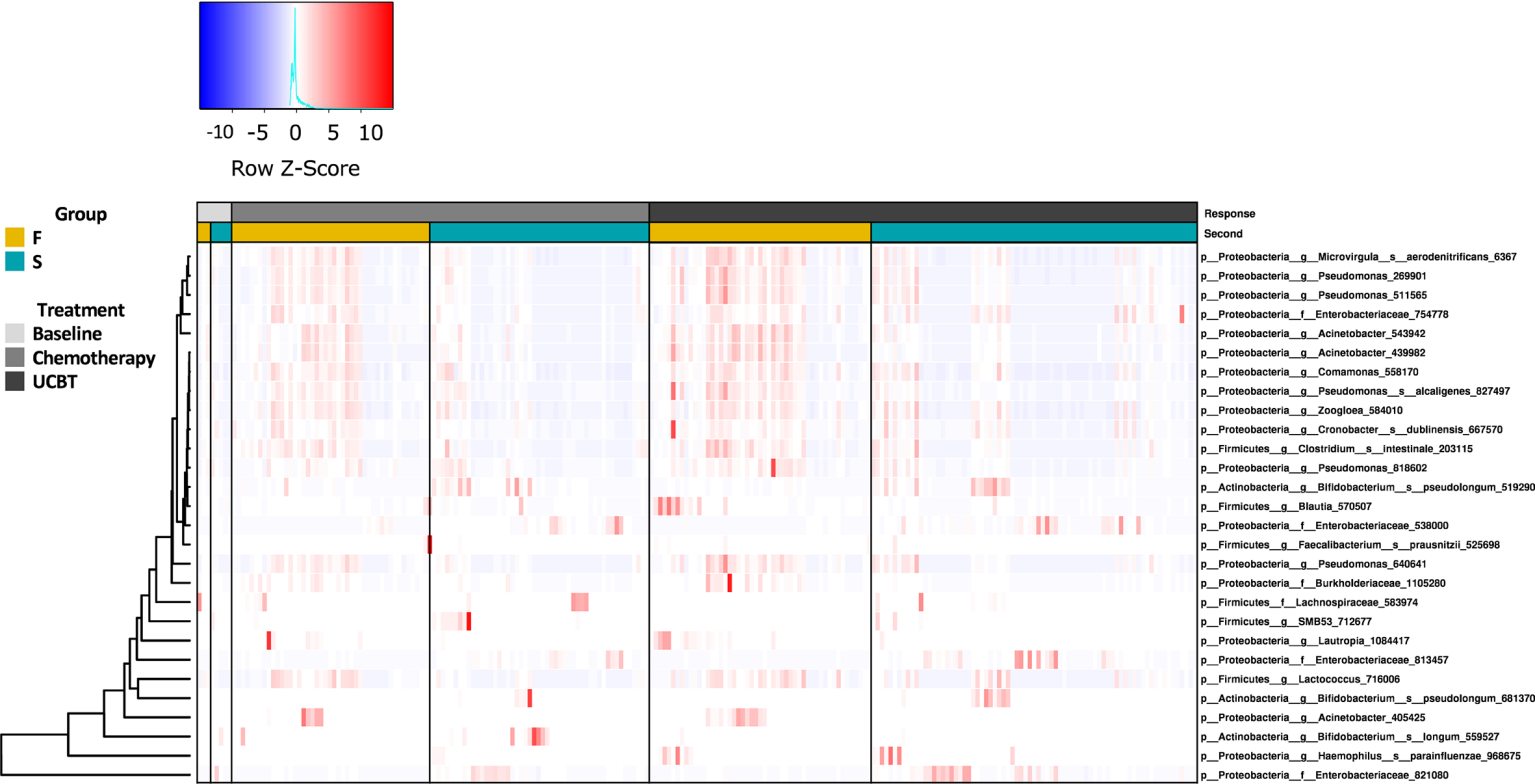
The relative abundance of bacterial genera in the S and F groups during the treatment course. Column represents samples from baseline, chemotherapy, and during UCBT.

Statistical analysis indicated the relative abundances of taxa between the F and S groups were highly significant (**Figure 2**). At baseline, there was no significant difference between the F and S group (**Table S2**). During the chemotherapy course, three taxa in the *Firmicutes* and eight in the *Proteobacteria* were enriched in the F group (**Table S3**). There were seven taxa more abundant in the S group during chemotherapy. Notably, there were relatively higher abundances of *Bifidobacterium longum*, *Bifidobacterium pseudolongum*, one taxon of the *Enterobacteriaceae* family (*Enterobacteriaceae_538000*), and one taxon of the *Lachnospiraceae* family in the S group during chemotherapy throughout the UCBT course (**Tables S3**, **S4**). Four taxa, including genera *Lautropia*, *Pseudomonas*, and species *Microvirgula aerodenitrificans*, were relatively higher abundances in the F group during chemotherapy throughout the UCBT (**Tables S3**, **S4**).

Next, we analyzed the organism-level microbiome phenotypes, including Gram staining, oxygen tolerance, ability to form biofilms, mobile element content, pathogenicity, and oxidative stress tolerance using BugBase. Prior to chemotherapy, there was no significant difference in these phenotype categories between the F and S groups (**Figure S5**). During chemotherapy, aerobic species were more abundant in the F group than that in the S group, which was likely attributed to a higher abundance of *Proteobacteria* (**Figure S6**). In contrast, higher abundances of facultatively anaerobic species and those capable of forming biofilms were found in the S group. During UCBT, most of the functional capacities, namely, facultative anaerobiosis, oxidative stress tolerance, mobile element content, biofilm formation, and pathogenesis, were lower in the F group than in the S group (**Figure S7**). However, the proportion of aerobic bacteria was still higher in the F group than in the S group after the UCBT.

### Gut Microbial Signatures Are Associated With Outcomes of UCBT Engraftment for Pediatric CD

**Figure 3:**
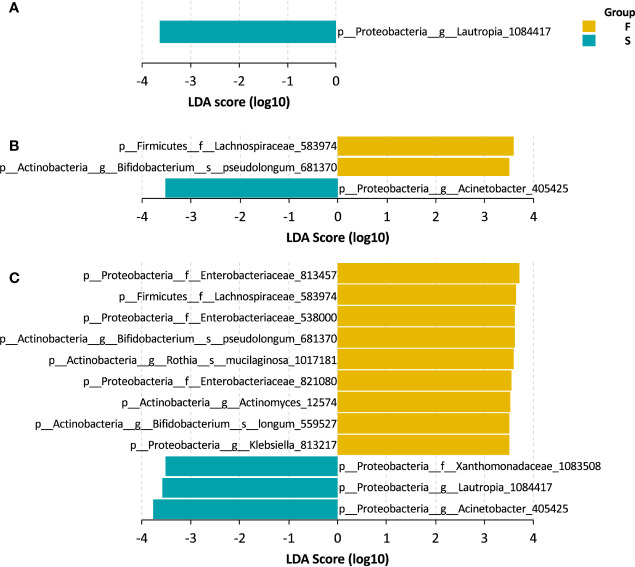
LEfSe bar of the representative taxa in the S and F groups during the treatment course. **(A)** Baseline. **(B)** Chemotherapy. **(C)** During UCBT.

We explored the gut bacteria that were closely associated with the outcomes of UCBT in pediatric CD patients. Using LEfSe, we identified several taxa that were representative of each group. At baseline, patients in the S group had a higher level of *Lautropia* genus that sustained throughout the UCBT (**Figures 3A, C**). After chemotherapy, a taxon of *Lachnospiraceae* family and *Bifidobacterium pseudolongum* were more abundant in the patients who failed the UCBT engraftment (**Figures 3B, C**). After UCBT, several taxa were found to be more prevalent in the F group after UCBT, including three OTUs of *Enterobacteriaceae* family, two species of *Bifidobacterium* genus, one OTU of *Lachnospiraceae* family, one OTU of *Rothia* genus, one OTU of *Actinomyces* genus, and one OTU of *Klebsiella* genus. In addition to *Lautropia*, a taxon assigned to *Acinetobacter* genus and another to *Xanthomonadaceae* family was more abundant in the S group after UCBT (**Figure 3C**).

**Figure 4:**
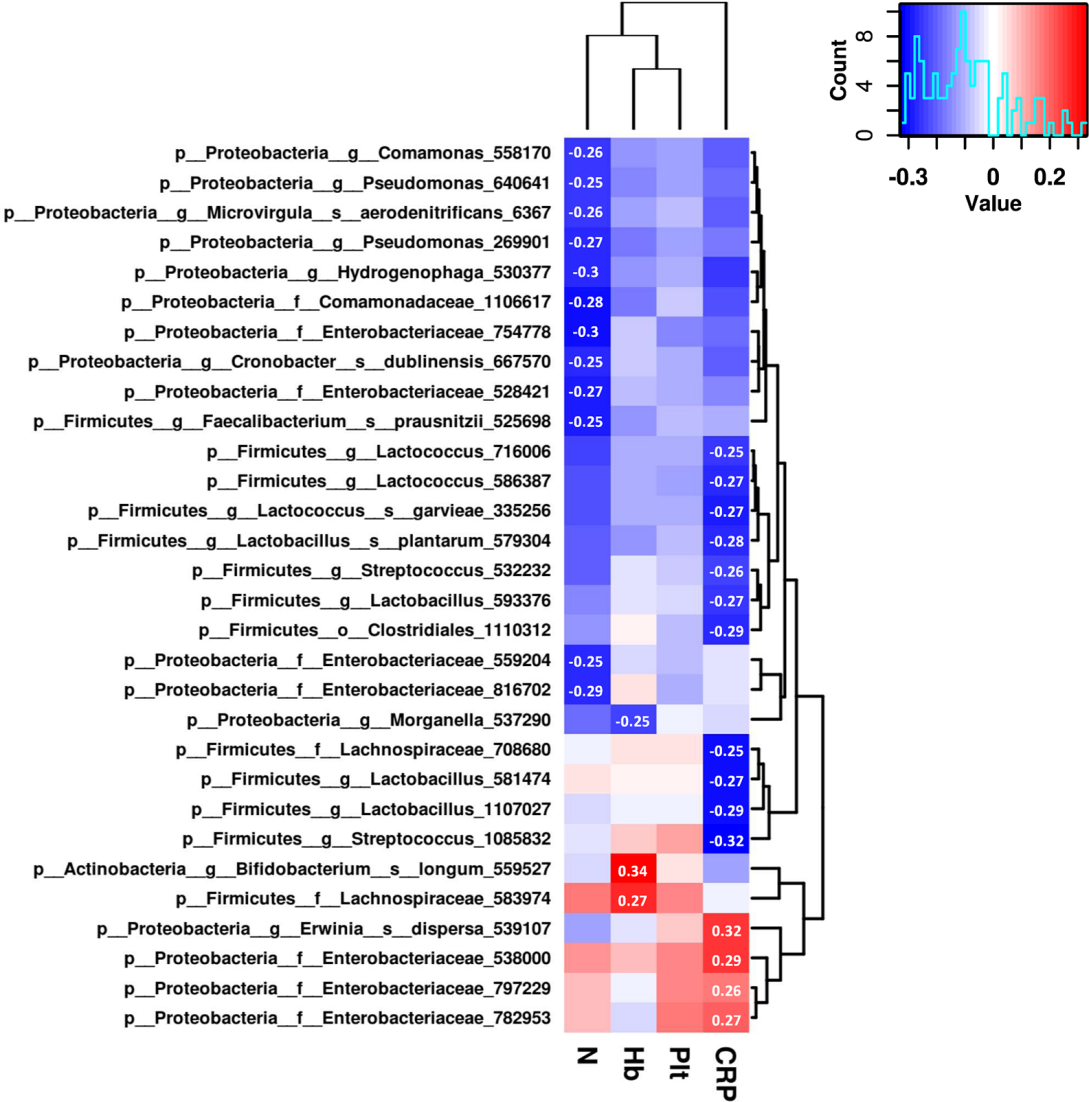
The correlation heat map shows correlations between clinical indices and the relative abundance of genera. Correlation coefficients with absolute values higher than 0.25 are shown in the blocks.

To further explore the associations of the gut microbiome and the therapeutic outcomes of UCBT, we analyzed the relative abundances of top 100 most abundant OTUs and serum levels of C-reactive protein, hemoglobin, neutrophil counts, and platelet counts by Spearman rank correlations. There were 30 OTUs significantly correlated (Spearman correlation index |R| ≥ 0.25 and P < 0.05) with four clinical indices (**Figure 4**). Specifically, C-reactive protein were positively correlated with the relative abundances of three taxa in the *Enterobacteriaceae* family, while negatively correlated with four taxa in the *Lactobacillus* genus and three taxa in the *Lactococcus* genus. The serum levels of hemoglobin were positively correlated with the relative abundances of *Bifidobacterium longum* and a taxon in the *Lachnospiraceae* family, while negatively correlated with a taxon in the *Morganella* genus. The neutrophil counts were negatively correlated with four taxa in the *Enterobacteriaceae* family. However, there was no statistical correlation between the relative abundances of gut bacteria and platelet counts in this population.

## Discussion

In this study, we identified that early intestinal microbial profiles, including a reduced diversity on the transplant day and during the chemotherapy, were associated with an engraftment failure in CD patients with *IL10RA* deficiency following the UCBT. To the best of our knowledge, there are a few studies on the dynamic changes in fecal microbiome during the UCBT in pediatric IBD patients with *IL10RA* deficiency. Our data suggest that the intestinal microbial profiles may help evaluating and managing the UCBT outcome in pediatric CD patients with *IL10RA* deficiency and those with primary immunodeficiency and severe malnutrition.

Previous studies have shown that allo-HSCT can change the diversity and stability of the intestinal flora (Taur et al., 2012; Taur et al., 2015). The reduction in microbial diversity and its related neutrophil engraftment failure are associated with increased risks of transplantation-related death (Peled and Gomes, 2020). In our study, we found that significantly reduced fecal microbial diversity was detected in those patients with engraftment failure on the day of transplant and the microbiome in those patients with engraftment failure had a greater variability in alpha-diversity and less heterogeneity in beta-diversity. Such data suggest that those patients may share a similar intestinal microbial community with lower resilience. However, we did not find a significant reduction in the microbial diversity post the UCBT in those patients, which may stem in part from the continued antibiotic pressure and RIC regimens.

A previous study observed that a single predominant bacterial taxon accounted for at least 30% of the microbiota in patients post allo-HSCT (Stein-Thoeringer and Nichols, 2019). Dramatically high abundance of *Enterococcus*, *Streptococcus*, or *Lactobacillus* was correlated with the severities of allo-HSCT-related complications. A high abundance of *Enterococci*, *especially E. faecium*, usually preceds the onset of GVHD, suggesting that intestinal dominant bacteria may exacerbate an intestinal and systemic inflammation (Shallis et al., 2018; Stein-Thoeringer and Nichols, 2019; Peled and Gomes, 2020). In this study, we observed that some patients (patient S4, S5, S6, S7, F1, and F3) developed GVHD and had dominant genera of *Klebsiella*, *Rothia*, *Enterococcus*, *Streptococcus*, and *Acinetobacter* in their fecal microbiota. We defined a bacterial domination as a genus with >30% of abundance in three consecutive samples after the UCBT. Previous studies also detected a high abundance of *Klebsiella* and *Streptococcus*, besides *Enterococcus* (Stein-Thoeringer and Nichols, 2019). Similarly, a high abundance of *Rothia* was found in fecal samples of infants with non-IgE-mediated cow milk protein allergy (Aparicio et al., 2020). We found that *Acinetobacter* was dominant in fecal microbiota of the F3 patient who died of sepsis, supporting the notion that many genera can cause substantial infections (Visca et al., 2011).

Both diversity metrics and signatures of specific bacterial abundance are valuable for evaluating healthy and disease status. The abundance of fecal *Bifidobacterium longum* was significantly correlated with blood hemoglobin levels in CD patients with *IL-10R*A deficiency following the UCBT. Moreover, the abundance of *Bifidobacterium longum* was significantly different between these two groups of patients during the chemotherapy and after the UCBT. We speculate that there may be a primary biological network of *Bifidobacterium* in the gut of patients (Luo et al., 2018). A previous study has shown that the *Bifidobacterium* genus benefits human health (Gigliucci et al., 2018). Additionally, the lack of beneficial *Bifidobacterium* species, together with other symbiont microbes, in the feces has been found in CD subjects, reflecting their probiotic nature (Alhagamhmad et al., 2016).

Multiple time points of intestinal microbiome during chemotherapy and post the UCBT were included for early detection of microbial configurations before platelet engraftment and neutrophil engraftment in this study. We found that the main difference in the diversity and abundance appeared to happen after the RIC and before the UCBT, which may reflect the variation in patients’ responses to the conditioning although all patients received the same RIC regimens. These indicate that longitudinal monitoring of intestinal microbiome is necessary for evaluating engraftment in CD patients with *IL-10R*A deficiency. Considering the variation of gut microbiome, ideal indicators for evaluating the engraftment failure may be the microbial characteristics which showed significant difference during chemotherapy and remained different throughout the UCBT course. Such observations suggest that more precise and personalized strategies are needed for the RIC in the management of CD patients. In addition, the variable qualities of donor UCB may also contribute to the changes in fecal microbiota in those patients after the UCBT. Although the UCBT is the best option for treatment of these patients because of its accessibility, the information of UCB donors usually is not available, which may be a challenge to precise personalized medicine for the UCBT of CD patients.

We recognized that this longitudinal observational study had several limitations. First, this study had a small sample size so that the microbial features observed in this exploratory study could only be laboratory diagnostic indicators for evaluating the outcome of transplantation currently. Although patients’ immune status is relatively uniform with the same *IL10RA* deficiency, the research findings need to be further verified in future study with a larger sample size. Consideration of other factors that may impact on microbial composition or the outcome of UCBT, including the condition of the donor and the quality of the UCB, should provide more clinical significance to find therapeutic biomarkers. Second, the descriptive nature of the study did not permit any causal inference. Because of these and the limited sample size, we could not determine whether these pioneer genera with prognostic potential impacted on host hematopoietic reconstitution or the alternative patterns of taxa were the consequences of early host immune recovery (e.g., regulation *via* microRNA) (Liu et al., 2016). Third, 16S rRNA sequencing had its limitations in interpreting the potential PICRUSt predictions. Newer methods, such as shotgun metagenomic sequencing, metabolomics, and metaproteomics, should be more valuable for a more comprehensive evaluation of the composition and functionality of human microbiome; species- or group-specific quantitative PCR or microarray approaches may facilitate the real-time clinical use of these microbial biomarkers and their validation in a bigger population.

In summary, we provided new insights into the early microbial biomarkers for predicting an engraftment failure in pediatric CD patients with *IL10R*A deficiency after the UCBT. Reduced diversity of microbiota and low abundance of *Bifidobacterium longum* whose abundance had significant positive correlation with blood hemoglobin levels might be good candidates of microbial biomarkers. If validated, these findings may be valuable for predicting and preventing engraftment failure in IBD patients following the allo-HSCT, particularly for those with deficiency in *XIAP* and *FOXP3*. We are interested in further investigating the precise biological mechanism and the clinical impact of these microbiota using new technologies. Potentially, these findings may develop new biomarkers in precociously predicting HSCT complications and guiding clinical intervention.

## Data Availability Statement

The data generated during the current study are available from the corresponding author on reasonable request.

## Ethics Statement

This study was approved by the Ethical Committee of Children’s Hospital, Fudan University (Shanghai, China, ethics no. [2017]-230). Written informed consent to participate in this study was provided by the participants’ legal guardian/next of kin.

## Author Contributions

YH was responsible for study design and supervision, drafting and critical revision of the manuscript. AX contributed to study design, acquisition and analysis of clinical data, and drafting and revision of manuscript. XQ and XG contributed to data collection and analysis and drafting the article. PW, LW, CZ, ZH, WH, and JS contributed to acquisition of samples and analysis of data. All authors contributed to the article and approved the submitted version.

## Conflict of Interest

The authors declare that the research was conducted in the absence of any commercial or financial relationships that could be construed as a potential conflict of interest.
