# Supplementary material for: Fecal Microbial Signatures Are Associated With Engraftment Failure Following Umbilical Cord Blood Transplantation in Pediatric Crohn’s Disease Patients With IL10RA Deficiency
Source: Front Pharmacol. 2020 Oct 8;11:580817. doi: 10.3389/fphar.2020.580817 (PMC7580494; doi:10.3389/fphar.2020.580817)
Supplement: Supplementary file 1 [file DataSheet_1.docx]

| **Table S1. Clinical information of IL10RA-deficiency patients receiving umbilical cord blood transplantation（UCBT）** | | | | | | | | | | | | | | |
| --- | --- | --- | --- | --- | --- | --- | --- | --- | --- | --- | --- | --- | --- | --- |
| **Pt** | **Age^1^ (m)** | **Gender** | **Weight^1^ (kg)** | **BMI^1^** | **CRP^1^ (mg/L)** | **Hb^1^ (g/L)** | **wPCDAI^1^** | **Duration^1^ (month)** | **Medications** | **Nutritional Support** | **IL10RA Gene Mutations** | **Platelets engraftment^2^** | **Neutrophil engraftment^2^** | **Clinical outcome** |
| S1 | 26 | M | 9.5 | 15.22 | ＜8 | 88 | 65 | 10 | MES, THD, Steroids | EN | c.C301T p.R101W/ c.C301T p.R101W | Day 19 | Day 31 | CR |
| S2 | 13 | F | 10 | 18.76 | 18 | 75 | 57.5 | 12 | MES, THD, Steroids | EN+PN | c.C301T p.R101W/ c.C301T p.R101W | Day 30 | Day 13 | CR |
| S3 | 5 | F | 5.6 | 14.42 | ＜8 | 97 | 37.5 | 5 | MES, THD | EN+PN | c.C301T p.R101W/ c.G537A p.T179 T | Day 25 | Day 29 | CR |
| S4 | 21 | M | 12 | 16.22 | 13 | 83 | 45 | 17 | MES, THD, PPI | EN+PN | c.G537A p.T179T/ c.569T.G p.F190.C | Day 17 | Day 21 | CR(with diffuse brain injury) |
| S5 | 5 | M | 3.7 | 11.8 | ＜8 | 93 | 12.5 | 4 | MES, THD, PPI | EN+PN | c.C301T p.R101W/ c.G537A p.T179T | Day 31 | Day 14 | CR |
| S6 | 17 | F | 7.5 | 16.22 | ＜8 | 101 | 45 | 17 | MES, THD | PN+EN | c.C301T p.R101W/ c.C301T p.R101W | Day 28 | Day 20 | CR |
| S7 | 13 | F | 5.5 | 12.25 | 18 | 104 | 20 | 12 | THD, Steroids | PN+EN | c.C301T p.R101W/ c.C301T p.R101W | Day 35 | Day 24 | CR |
| F1 | 9 | M | 10 | 19.84 | ＜8 | 133 | 5 | 9 | MES, THD | PN+EN | c.C301T p.R101W/ c.G537A p.T179T | NA | NA | Died (respiratory failure) |
| F2 | 14 | M | 7.5 | 16.71 | 11 | 99 | 47.5 | 14 | MES, THD, Steroids | PN+EN | c.T299G p.V100G/ c.G99A p.W33X | NA | NA | Died (respiratory failure) |
| F3 | 8 | F | 6.2 | 13.81 | 23 | 93 | 37.5 | 8 | MES, THD | PN+EN | c.C301T p.R101W/ c.C301T p.R101W | NA | NA | Died (sepsis) |
| F4 | 7 | M | 5 | 14.86 | ＜8 | 101 | 35 | 6 | MES, THD | PN+EN | c.T299G p.V100G/c.C301T p.R101W | NA | NA | Died (sepsis) |
| F5 | 27 | M | 6 | 15.61 | 24 | 91 | 55 | 26 | MES, THD | PN+EN | c.C301T p.R101W/c.C349T p.R117C | NA | NA | Awaiting for the second transplant |
| F6 | 28 | F | 11 | 16.77 | ＜8 | 86 | 37.5 | 28 | MES, THD | PN+EN | c.C301T p.R101W/ c.G537A p.T179T | NA | NA | Died (sepsis) |

^1^clinical variables at transplantation; ^2^days post transplant.

Abbreviations: Pt, Patient; Hb, Hemoglobin; MES, mesalazine; THD, thalidomide; PN, parenteral nutrition; EN, enteral nutrition; NA, not applicable; CR, complete remission.

**Table S2. Comparisons of relative abundance of taxa between F and S groups at baseline**

| **Taxa** | **Wilcoxon**  **rank sum test P** | **Bonferroni**  **adjusted P** | **F**  **Mean RA%** | **S**  **Mean RA%** |
| --- | --- | --- | --- | --- |
| **Higher in F group** |  |  |  |  |
| p__Firmicutes__g__SMB53_712677 | 0.036 | 1 | 0.073 | 0.67 |
| p__Firmicutes__g__Clostridium_555562 | 0.036 | 1 | 0.21 | 0.59 |
| p__Firmicutes__f__Clostridiaceae_843459 | 0.036 | 1 | 0.18 | 0.12 |
| p__Firmicutes__g__Clostridium__s__intestinale_203115 | 0.036 | 1 | 0.13 | 0.046 |
| p__Proteobacteria__g__Lautropia_1084417 | 0.036 | 1 | 0.15 | 0.018 |
| p__Proteobacteria__g__Pseudomonas_511565 | 0.036 | 1 | 0.33 | 0.084 |

RA: relative abundance.

**Table S3. Comparisons of relative abundance of genera between F and S groups during chemotherapy**

| **Taxa** | **Wilcoxon**  **rank sum test P** | **Bonferroni**  **adjusted P<0.01** | **F**  **Mean RA%** | **S**  **Mean RA%** |
| --- | --- | --- | --- | --- |
| **Higher in F group** |  |  |  |  |
| p__Firmicutes__g__Faecalibacterium__s__prausnitzii_525698 | 0.00002 | 0.002 | 0.2 | 0.015 |
| p__Firmicutes__g__Clostridium__s__intestinale_203115 | 0.000077 | 0.0077 | 0.2 | 0.072 |
| p__Firmicutes__g__Lactococcus_716006 | 0.000052 | 0.0052 | 4.01 | 1.22 |
| p__Proteobacteria__g__Lautropia_1084417 | 0.000066 | 0.0066 | 1.43 | 0.054 |
| p__Proteobacteria__g__Cronobacter__s__dublinensis_667570 | 0.000000014 | 0.0000014 | 0.15 | 0.049 |
| p__Proteobacteria__f__Burkholderiaceae_1105280 | 0.0000077 | 0.00077 | 0.73 | 0.16 |
| p__Proteobacteria__g__Microvirgula__s__aerodenitrificans_6367 | 0.0000091 | 0.00091 | 0.39 | 0.13 |
| p__Proteobacteria__g__Pseudomonas_269901 | 0.000043 | 0.0043 | 0.35 | 0.11 |
| p__Proteobacteria__g__Pseudomonas_640641 | 0.000058 | 0.0058 | 1.31 | 0.43 |
| p__Proteobacteria__f__Enterobacteriaceae_754778 | 0.0000013 | 0.00013 | 0.78 | 0.3 |
| p__Proteobacteria__f__Enterobacteriaceae_813457 | 0.000000036 | 0.0000036 | 0.19 | 1.86 |
|  |  |  |  |  |
| **Higher in S group** |  |  |  |  |
| p__Actinobacteria__g__Bifidobacterium__s__longum_559527 | 0.000011 | 0.0011 | 0.53 | 4.46 |
| p__Actinobacteria__g__Bifidobacterium__s__pseudolongum_519290 | 0.000031 | 0.0031 | 0.035 | 0.1 |
| p__Actinobacteria__g__Bifidobacterium__s__pseudolongum_681370 | 0.000000023 | 0.0000023 | 0.0041 | 1.24 |
| p__Firmicutes__f__Lachnospiraceae_583974 | 0.00000059 | 0.000059 | 0.42 | 1.45 |
| p__Firmicutes__g__SMB53_712677 | 0.0000078 | 0.00078 | 0.15 | 1.15 |
| p__Proteobacteria__f__Enterobacteriaceae_538000 | 0.0000036 | 0.00036 | 0.069 | 0.24 |
| p__Proteobacteria__g__Haemophilus__s__parainfluenzae_968675 | 0.00000013 | 0.000013 | 0.19 | 0.65 |

RA: relative abundance.

**Table S4. Comparisons of relative abundance of taxa F and S groups during UCBT**

| **Taxa** | **Wilcoxon**  **Rank Sum Test P** | **Bonferroni**  **adjusted P<0.01** | **F**  **Mean RA%** | **S**  **Mean RA%** |
| --- | --- | --- | --- | --- |
| **Higher in F group** |  |  |  |  |
| p__Firmicutes__g__Blautia_570507 | 0.00000026 | 0.000026 | 0.11 | 0.014 |
| p__Proteobacteria__g__Acinetobacter_405425 | 0.0000032 | 0.00032 | 3.4 | 0.0039 |
| p__Proteobacteria__g__Lautropia_1084417 | 0.0000073 | 0.00073 | 1.62 | 0.11 |
| p__Proteobacteria__g__Pseudomonas__s__alcaligenes_827497 | 0.00000054 | 0.000054 | 0.11 | 0.023 |
| p__Proteobacteria__g__Acinetobacter_439982 | 0.0000033 | 0.00033 | 0.11 | 0.023 |
| p__Proteobacteria__g__Comamonas_558170 | 0.00000018 | 0.000018 | 0.076 | 0.032 |
| p__Proteobacteria__f__Burkholderiaceae_1105280 | 3.6E-09 | 0.00000036 | 1.27 | 0.15 |
| p__Proteobacteria__g__Zoogloea_584010 | 0.0000011 | 0.00011 | 0.13 | 0.065 |
| p__Proteobacteria__g__Pseudomonas_818602 | 0.0000019 | 0.00019 | 0.26 | 0.088 |
| p__Proteobacteria__g__Pseudomonas_511565 | 0.000000084 | 0.0000084 | 0.67 | 0.23 |
| p__Proteobacteria__g__Pseudomonas_269901 | 4.3E-09 | 0.00000043 | 0.48 | 0.16 |
| p__Proteobacteria__g__Microvirgula__s__aerodenitrificans_6367 | 0.00000058 | 0.000058 | 0.47 | 0.17 |
| p__Proteobacteria__g__Acinetobacter_543942 | 0.00000026 | 0.000026 | 1.35 | 0.35 |
| p__Proteobacteria__g__Pseudomonas_640641 | 0.00000026 | 0.000026 | 1.75 | 0.67 |
|  |  |  |  |  |
| **Higher in S group** |  |  |  |  |
| p__Actinobacteria__g__Bifidobacterium__s__longum_559527 | 0.00000019 | 0.000019 | 0.13 | 0.28 |
| p__Actinobacteria__g__Bifidobacterium__s__pseudolongum_681370 | 0.0000024 | 0.00024 | 0.0035 | 1.29 |
| p__Firmicutes__f__Lachnospiraceae_583974 | 1.8E-13 | 1.8E-11 | 0.016 | 0.58 |
| p__Proteobacteria__f__Enterobacteriaceae_821080 | 0.0000078 | 0.00078 | 3.03 | 14.17 |
| p__Proteobacteria__f__Enterobacteriaceae_813457 | 6.5E-14 | 6.5E-12 | 0.17 | 2.78 |
| p__Proteobacteria__f__Enterobacteriaceae_538000 | 5.8E-11 | 5.8E-09 | 0.031 | 0.26 |

RA: relative abundance.
