# Supplementary material for: Fecal Microbial Signatures Are Associated With Engraftment Failure Following Umbilical Cord Blood Transplantation in Pediatric Crohn’s Disease Patients With IL10RA Deficiency
Source: Front Pharmacol. 2020 Oct 8;11:580817. doi: 10.3389/fphar.2020.580817 (PMC7580494; doi:10.3389/fphar.2020.580817)
Supplement: Supplementary file 2 [file DataSheet_2.pdf]

Supplementary Tables & Figures

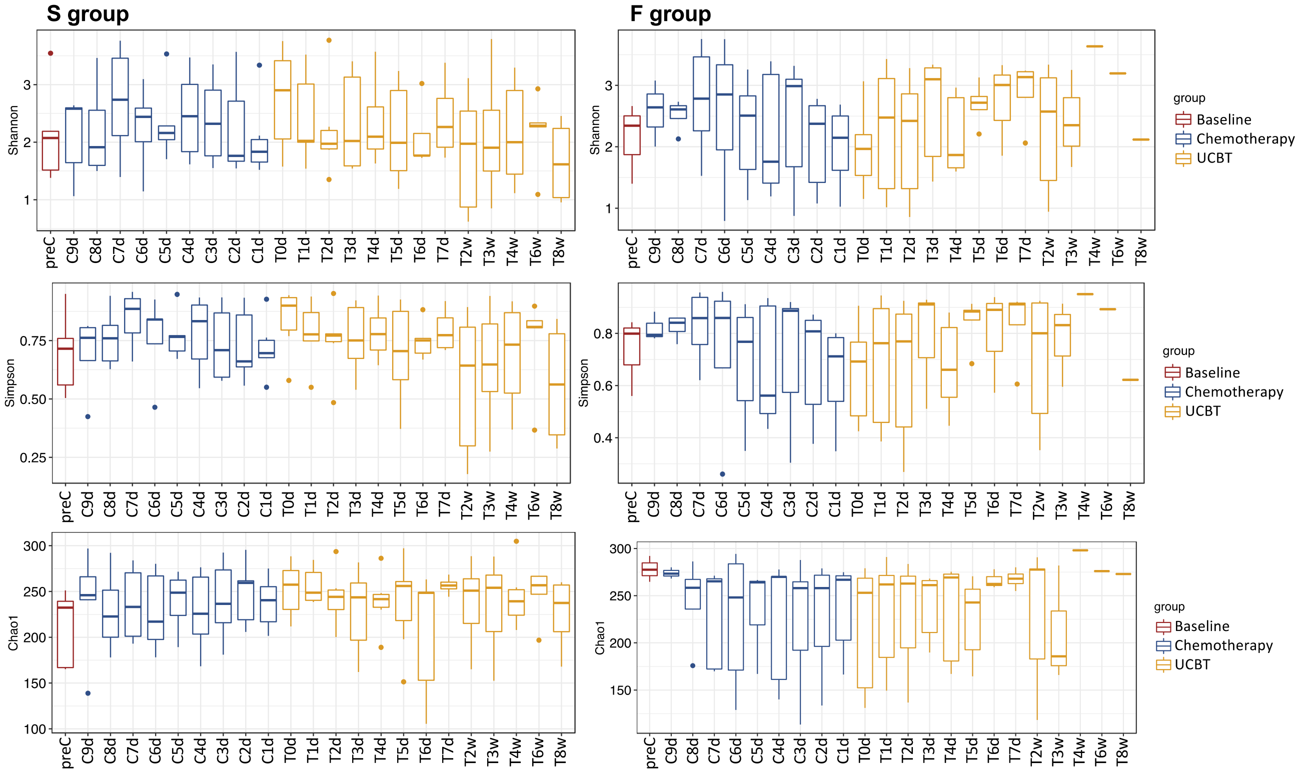

Figure S1. The dynamic changes in the alpha diversity of gut microbiota in the F and S groups of patients. The alpha diversity of gut microbiota was measured with (**A**) Shannon index, (**B**) Chao1 index, and (**C**) Simpson index over the screening period.

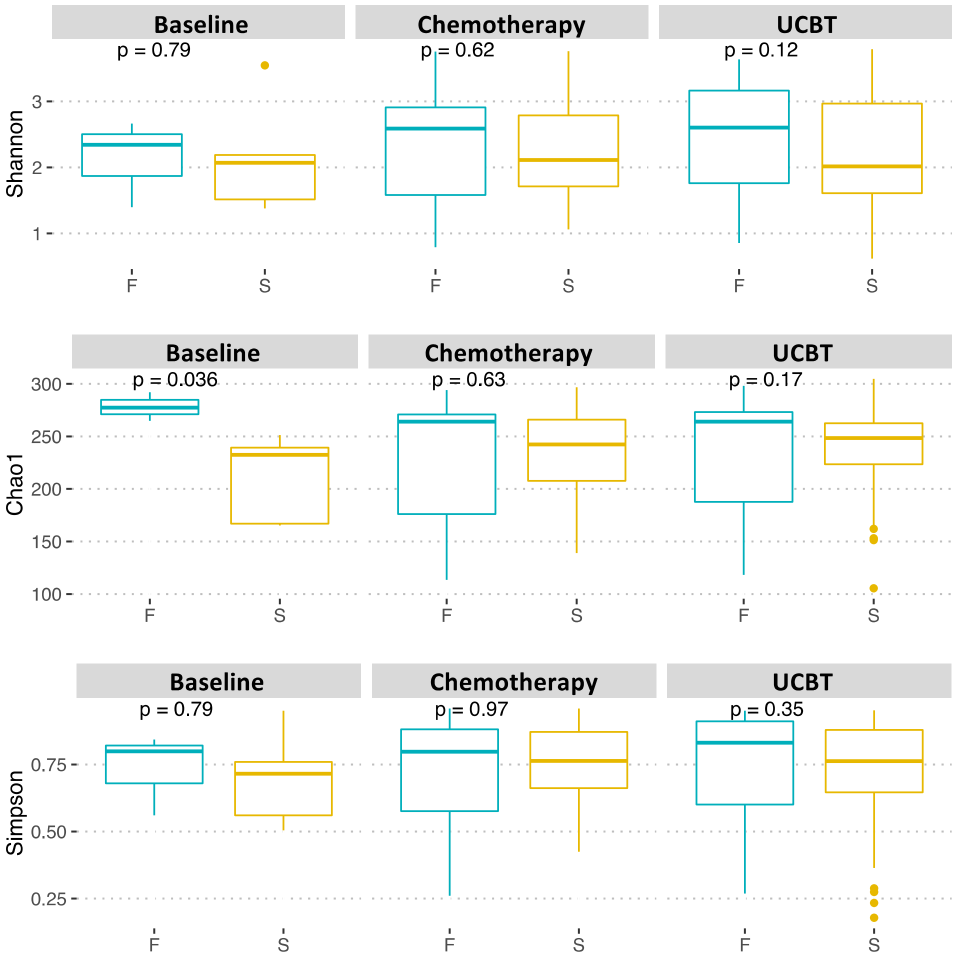

Figure S2. Comparisons of the alpha diversity in fecal microbiota between the F and S groups during the treatment course. The alpha-diversity was measured with (**A**) Shannon's, (**B**) Chao1's, and (**C**) Simpson's indices.

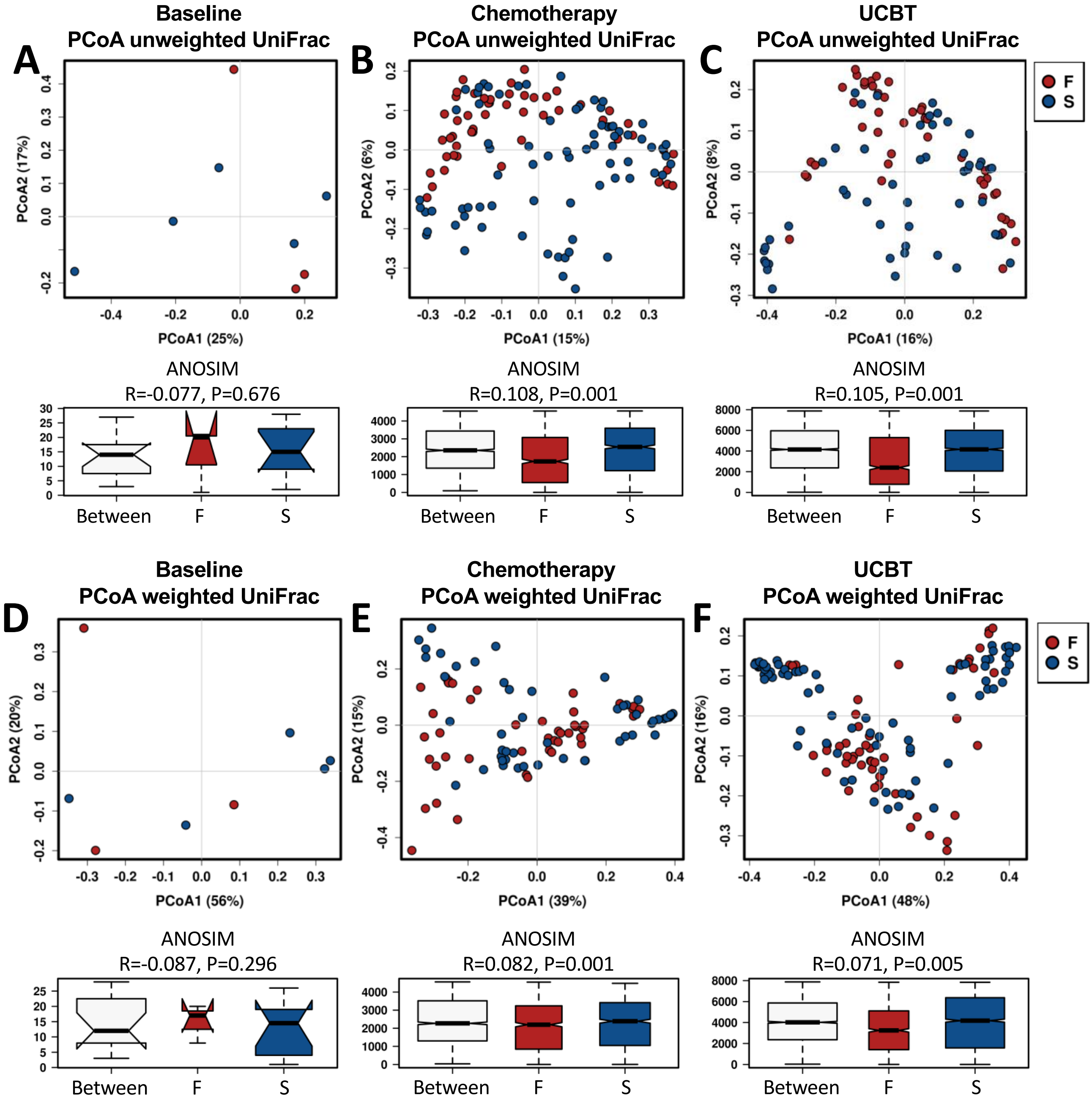

Figure S3. PCoA analysis of the beta-diversity differences based on unweighted and weighted UniFrac distances. Samples of the F and G groups were colored red and blue, respectively, and were compared at (**A, D**) baseline, (**B, E**) during chemotherapy, and (**C, F**) during UCBT.

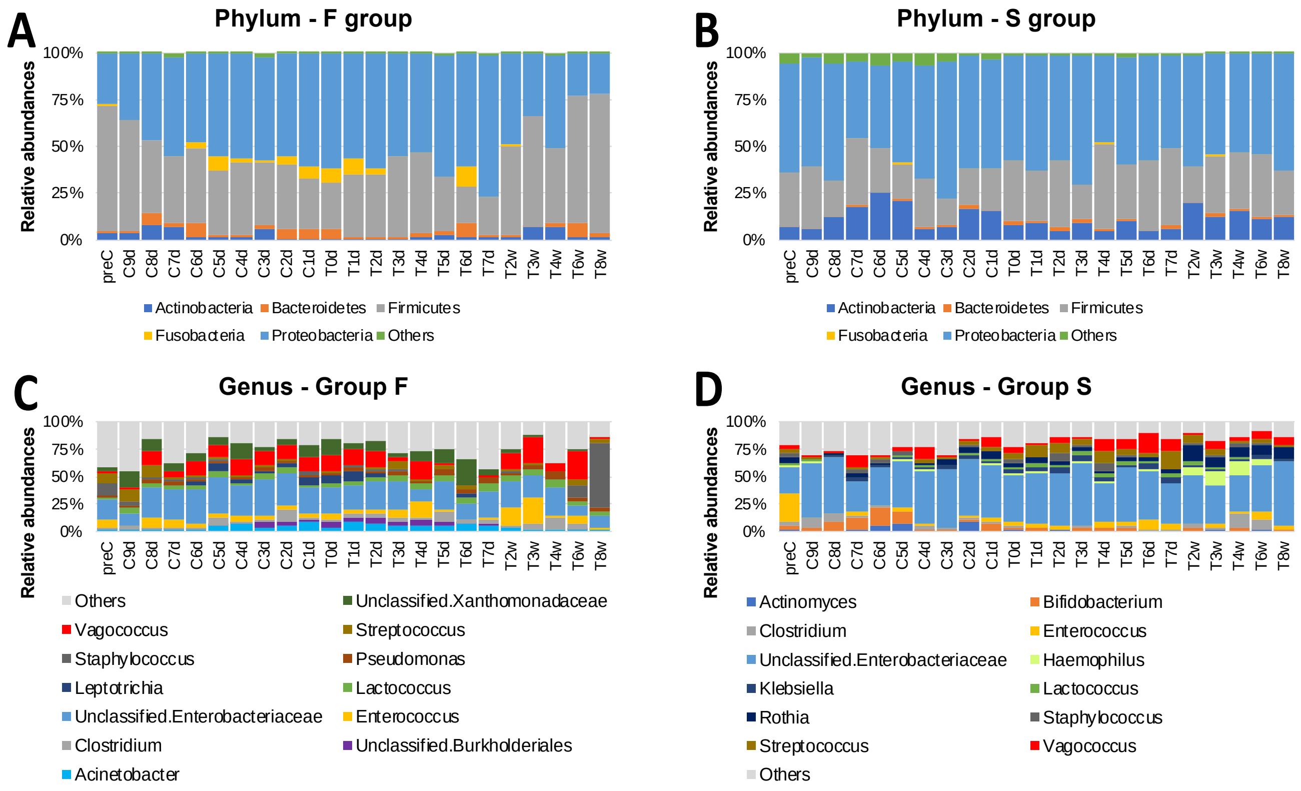

Figure S4. The dominant gut bacterial taxa in the F and S groups. The relative abundances of the most prevalent (**A, B**) Phylum and (**C, D**) genera in each group.

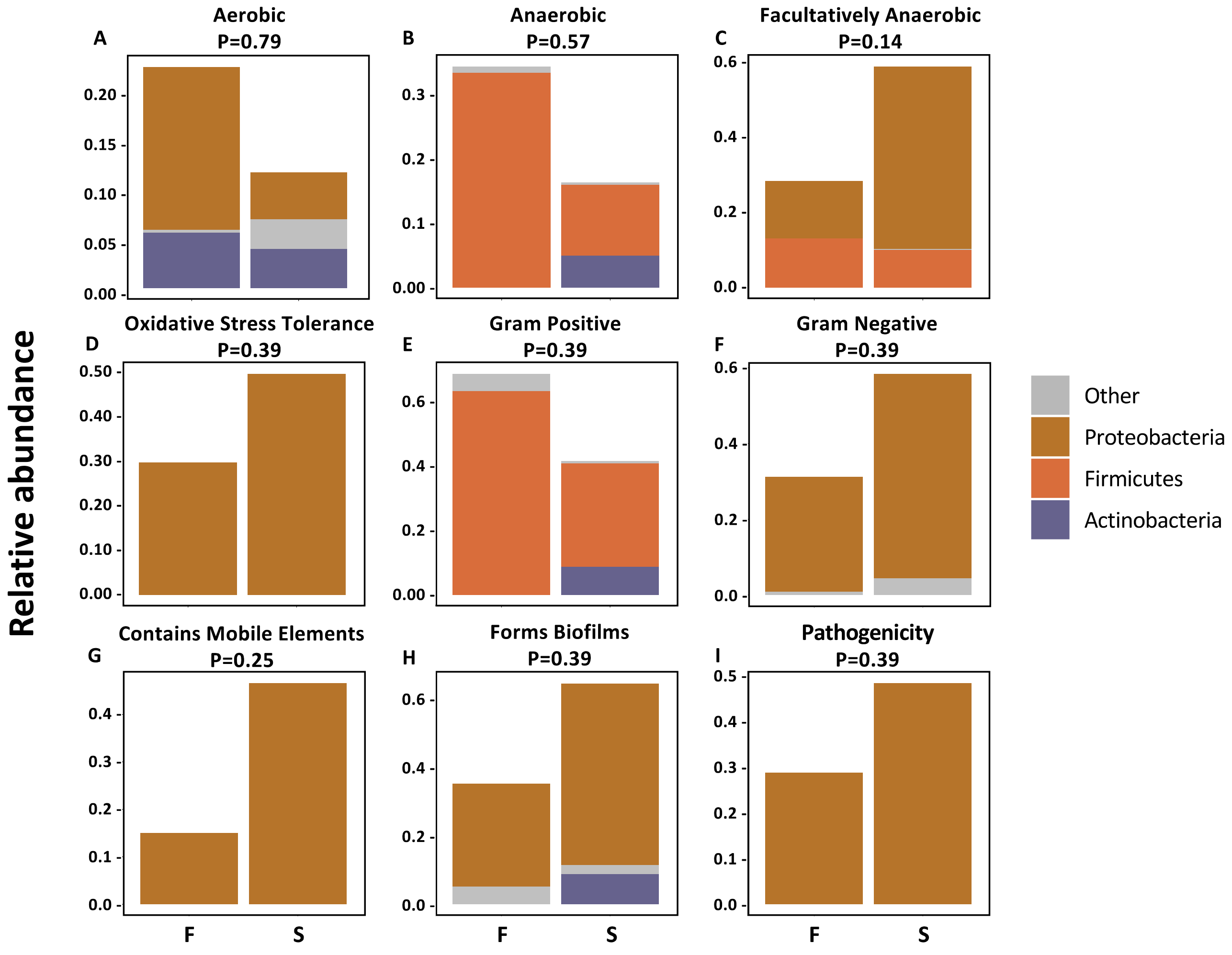

Figure S5. Organism-level microbiome phenotypes in the F and S groups at baseline. Bubase was used to access the relative abundances of **(A)** aerobic bacteria, **(B)** anaerobic bacteria, **(C)** facultatively anaerobic bacteria, **(D)** oxidative stress tolerance, **(E)** Gram-negative bacteria, **(F)** Gram-positive bacteria, **(G)** mobile element content, **(H)** biofilm formation, and **(I)** pathogenesis.

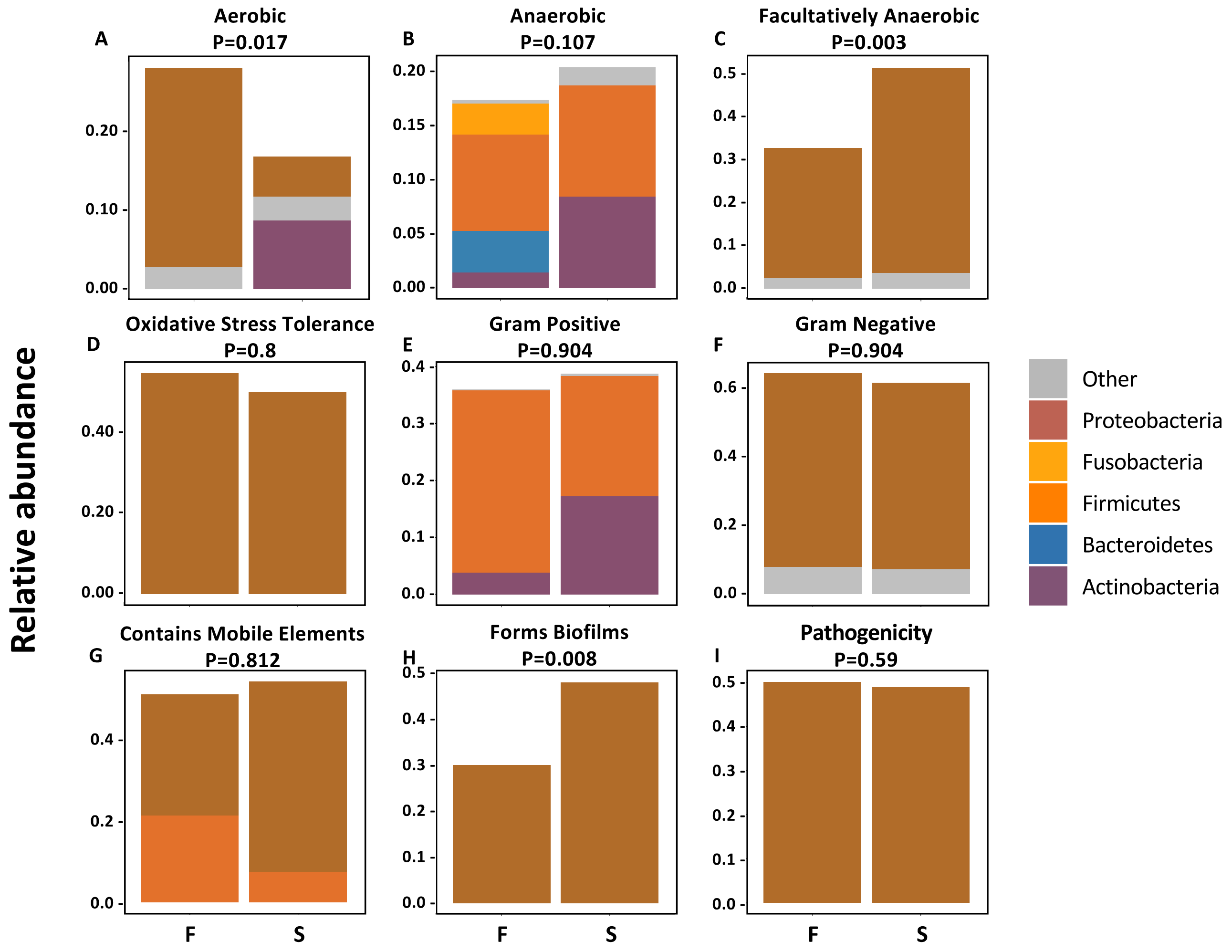

Figure S6. Organism-level microbiome phenotypes in the F and S groups during chemotherapy. Bubase was used to access the relative abundances of **(A)** aerobic bacteria, **(B)** anaerobic bacteria, **(C)** facultatively anaerobic bacteria, **(D)** oxidative stress tolerance, **(E)** Gram-negative bacteria, **(F)** Gram-positive bacteria, **(G)** mobile element content, **(H)** biofilm formation, and **(I)** pathogenesis.

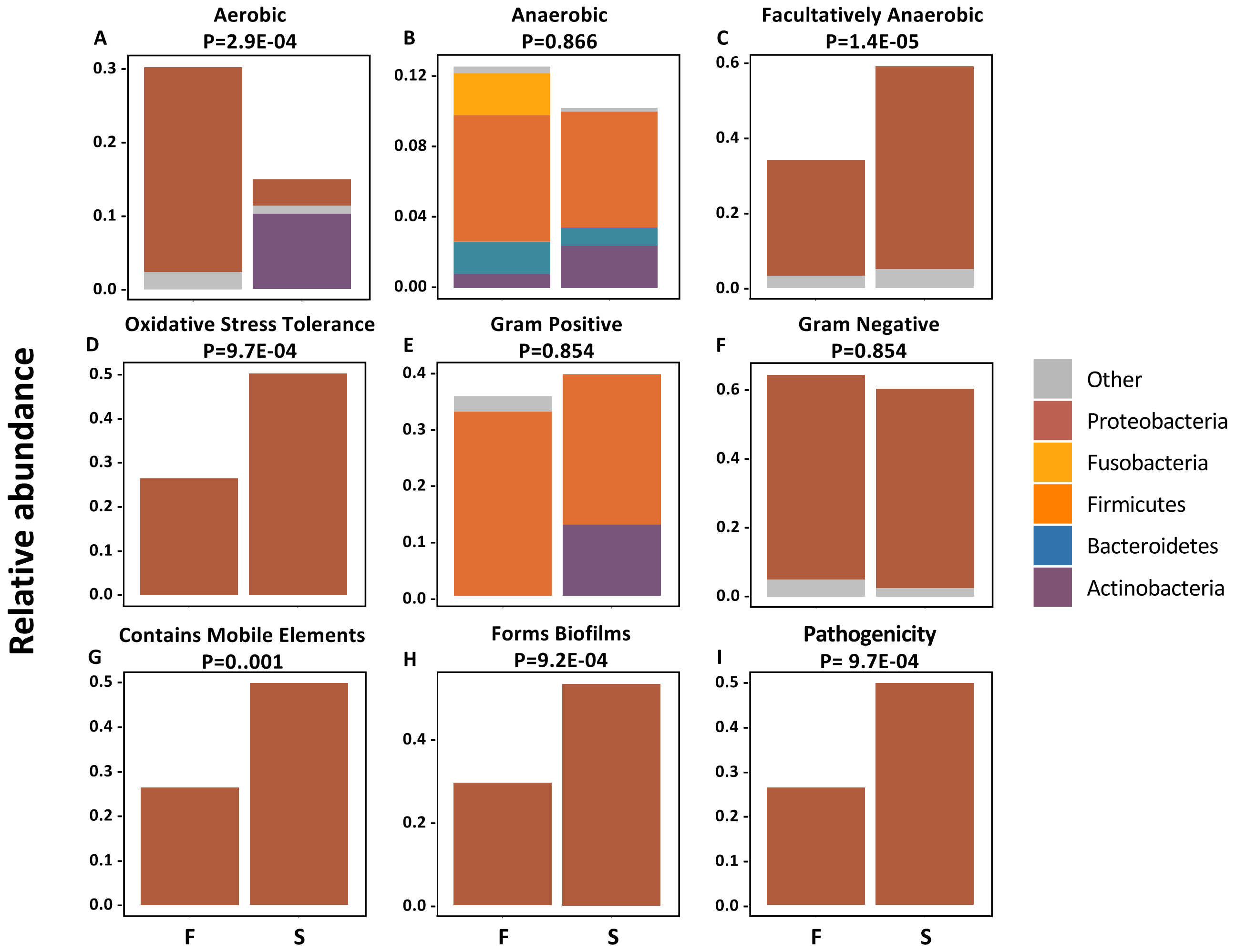

Figure S7. Organism-level microbiome phenotypes in the F and S groups during UCBT. Bubase was used to access the relative abundances of **(A)** aerobic bacteria, **(B)** anaerobic bacteria, **(C)** facultatively anaerobic bacteria, **(D)** oxidative stress tolerance, **(E)** Gram-negative bacteria, **(F)** Gram-positive bacteria, **(G)** mobile element content, **(H)** biofilm formation, and **(I)** pathogenesis.
